# Antimicrobial resistance of *Neisseria gonorrhoeae* in Germany: low levels of cephalosporin resistance, but high azithromycin resistance

**DOI:** 10.1186/s12879-018-2944-9

**Published:** 2018-01-17

**Authors:** Susanne Buder, Sandra Dudareva, Klaus Jansen, Anna Loenenbach, Sergejs Nikisins, Andrea Sailer, Eva Guhl, Peter K. Kohl, Viviane Bremer, GORENET study group, Thomas Back, Anja Berger, Valerie Chapot, Jörg Steinmann, Stephan Eicke, Claudia Friedrichs, Andreas Groß, Hans-Jochen Hagedorn, Alexander Halfmann, Britt Hornei, Ralf Ignatius, Simone Korten, Hany Sahly, Elzbieta Kozub-Witkowski, Sabine Krämer, Margit Kühn, Anke Liebetrau, Thomas Meyer, Klaus Oberdorfer, Roland Pfüller, Caroline Ruckert, Roman Schwarz, Daniela Walch, Madeleine Mai, Thomas A. Wichelhaus, Hilmar Wisplinghoff, Nicole Wüppenhorst

**Affiliations:** 1 German Consiliary Laboratory for Gonococci, Department of Dermatology and Venerology, Vivantes Hospital Berlin, Berlin, Germany; 2 Department for Infectious Disease Epidemiology, Robert Koch Institute, Berlin, Germany; 3 Charité University Medicine Berlin, Berlin, Germany; 4 Department of Infectious Diseases, Robert Koch Institute, Berlin, Germany; 5 European Public Health Microbiology Training (EUPHEM) programme, European Centre for Disease Prevention and Control (ECDC), Stockholm, Sweden

**Keywords:** *Neisseria gonorrhoeae*, Gonorrhoea, Antimicrobial resistance, Resistance surveillance

## Abstract

**Background:**

The widespread antimicrobial resistance of *Neisseria gonorrhoeae* is a serious problem for the treatment and control of gonorrhoea. Many of the previously effective therapeutic agents are no longer viable. Because *N. gonorrhoeae* infections are not reportable in Germany, only limited data on disease epidemiology and antimicrobial susceptibility patterns are available. The Gonococcal Resistance Network (GORENET) is a surveillance project to monitor trends in the antimicrobial susceptibility of *N. gonorrhoeae* in Germany in order to guide treatment algorithms and target future prevention strategies.

**Methods:**

Between April 2014 and December 2015, data on patient-related information were collected from laboratories nationwide, and susceptibility testing was performed on 537 *N. gonorrhoeae* isolates forwarded from the network laboratories to the Conciliar Laboratory for gonococci. Susceptibility results for cefixime, ceftriaxone, azithromycin, ciprofloxacin and penicillin were defined according to EUCAST 4.0 standards. Percentages, medians and interquartile ranges (IQR) were calculated.

**Results:**

Altogether, 90% of isolates were from men. The median age was 32 (IQR 25–44) years for men and 25 (IQR 22–40) years for women (*p*-value < 0.001). The most frequently tested materials among men were urethral (96.1%) and rectal swabs (1.7%), and among women, it was mainly endocervical and vaginal swabs (84.3%). None of the isolates were resistant to ceftriaxone. Furthermore, 1.9% (in 2014) and 1.4% (in 2015) of the isolates were resistant to cefixime, 11.9% and 9.8% showed resistance against azithromycin, 72.0% and 58.3% were resistant to ciprofloxacin, and 29.1% and 18.8% were resistant to penicillin**.**

**Conclusions:**

Resistance to ceftriaxone was not detected, and the percentage of isolates with resistance to cefixime was low, whereas azithromycin resistance showed high levels during the observation period. The rates of ciprofloxacin resistance and penicillin resistance were very high across Germany. Continued surveillance of antimicrobial drug susceptibilities for *N. gonorrhoeae* remains highly important to ensure efficient disease management.

## Background

The worldwide development of antimicrobial resistance in *Neisseria gonorrhoeae* is a serious problem for the treatment and control of gonorrhoea. Treatment opportunities are dramatically limited because many of the previously recommended therapeutic agents are no longer effective.

The World Health Organization (WHO) [1], the Centers for Disease Control and Prevention (CDC) [2], and the European Centre for Disease Prevention and Control (ECDC) [3] have called for action to confine the spread of multidrug-resistant *N. gonorrhoeae* by enhancing the surveillance system of *N. gonorrhoeae* susceptibility testing and by strengthening laboratory capacities to perform culture and antimicrobial resistance testing. Representative coverage of collected data, unified *N. gonorrhoeae* susceptibility testing methods and interpretation standards are of great importance [4–6].

Extended spectrum cephalosporins (ESCs) are the last agents effective against *N. gonorrhoeae*. However, resistance to ESCs is increasingly common [7–13], causing concern that the efficacy of this substance group could expire in the near future [5–10]. Due to the worldwide rising resistance of *N. gonorrhoeae* against cefixime in recent years, the treatment guidelines needed to be changed accordingly. Thus, cefixime is no longer recommended as first-line therapy in many countries [14–16] and new therapy guidelines and action plans have been developed to keep gonorrhoea a treatable disease. In 2014 the German STI Society implemented new therapy guidelines for Germany. These guidelines recommend the use of the ESC ceftriaxone (1000 mg single dose i.m. or i.v.), now in combination with azithromycin as dual therapy (1.5 g single dose p.o.), as the first-line treatment [17]. The use of cefixime (800 mg single dose p.o.) should be reserved for cases where parenteral treatment is not possible and ideally after susceptibility testing.

Because gonorrhoea is not a notifiable disease in Germany, only very limited data about epidemiology and antimicrobial resistance against *N. gonorrhoeae* are available. Furthermore, no standard operating procedures or unified protocols for *N. gonorrhoeae* susceptibility testing have been established in Germany. To date, information about *N. gonorrhoeae* susceptibility can be derived from several cross-sectional, regionally limited studies [18–21] through the German antibiotic resistance surveillance programme (ARS) and 100–120 isolates submitted to Euro-GASP yearly through the Consiliary Laboratory (CL) for gonococci [22]. Within this Euro-GASP collection, isolates resistant against ceftriaxone (MIC >0.125 mg/L) have been observed in Germany. Resistance against ceftriaxone was shown by 6.5% (*n* = 7) of all German Euro-GASP isolates in 2011, while in the following years 2012–2014, one isolate per year (1%) could be identified with ceftriaxone resistance. Cefixime resistance (MIC >0.125 mg/L) in German Euro-GASP isolates ranged between 5.7% and 12.9% (*n* = 13, 11.9% in 2010; *n* = 11, 10.2% in 2011; *n* = 6, 5.7% in 2012; *n* = 13, 12.9% in 2013) [22]. In 2014, there was no detection of cefixime-resistant isolates in Euro-GASP.

To implement continuous routine data collection on epidemiology and antimicrobial susceptibility testing for *N. gonorrhoeae* in German laboratories (aim 1) and to collect isolates for testing in the German CL for gonococci with unified methodology (aim 2), we set up a *N. gonorrhoeae* resistance network (GORENET).

We analysed the data and isolates collected through GORENET in 2014 and 2015 to guide treatment algorithms and targeted prevention strategies in Germany.

## Methods

To characterise laboratories testing for *N. gonorrhoeae* in Germany and indicate laboratories for recruitment, we performed a cross-sectional survey between June and August 2013, as described previously [23]. From the laboratories that expressed an interest in participating, we recruited private and hospital laboratories for GORENET, prioritizing those with a wider catchment area and a higher number of *N. gonorrhoeae* tests per quarter. The laboratories in Germany have no predefined catchment areas, and practitioners are free to choose laboratories for cooperation. For a better geographical coverage of the data, we strove to recruit laboratories from all regions in Germany and laboratories that use any gradient Etest for *N. gonorrhoeae* susceptibility testing, at least for azithromycin, ceftriaxone and cefixime. Participation was voluntary, and there was no financial compensation for laboratories to participate in the study. The data collection protocol was confirmed by the data protection officer at the Robert Koch Institute (RKI), Berlin. Additional approval from an ethics committee was deemed to be unnecessary.

### Continuous routine data collection

From GORENET network laboratories, continuous routine data on all samples tested for *N. gonorrhoeae* antimicrobial susceptibility were collected between April 2014 and December 2015. The network laboratories submitted data to the RKI (further labelled as samples). The collected data included sample identification number, information on test results, sampled material (urethral swab, urine, vaginal swab, cervical swab, rectal swab, pharyngeal swab, and other material), date of sampling, date of testing, district code, gender and year of birth. If the district code of the patient was not available, we used the code of the laboratory instead. Based on the year of birth, we calculated the person’s age at the time of sampling. If the date of testing was not available, we used the date when the isolate was received in the CL.

Data were transmitted electronically to the RKI. Laboratories entered the data either in an online questionnaire (VOXCO Command Center 3) or in a preformatted Excel spreadsheet (.xls). Data on *N. gonorrhoeae* susceptibility from laboratories willing to participate in GORENET but already submitting their susceptibility data (including data on a wide range of other agents) to the German Antibiotic Resistance Surveillance Programme (ARS) were extracted from the ARS database. We performed plausibility checks on all reported data.

Based on the district codes, we described the geographical distribution of the samples tested for *N. gonorrhoeae* antimicrobial susceptibility. Each district code or respective 3-digit postal code corresponded to one district in Germany. We used samples tested for susceptibility from participating laboratories to describe tested persons by gender, age, sampled material and treating specialist. Susceptibility results from participating laboratories are used for national surveillance, but not presented in this paper.

An overview of the analysis of samples tested for *N. gonorrhoeae* susceptibility in network laboratories is given in Table 1.

**Table 1:** Isolates tested for susceptibility in network laboratories and in CL, number of laboratories and isolates included in analysis and description of performed analysis for each data source

	Number of laboratories	Number of samples or isolates			In manuscript referred as	Performed analysis
		Total	2014	2015		
Samples tested for *N. gonorrhoeae* susceptibility in network laboratories	23	1654	727	927	Samples	Geographical, age and gender distribution. Sampled material and treating specialist
Isolates tested in CL	16	537	261	276	Isolates	Susceptibility for ceftriaxone, cefixime, azithromycin, penicillin and ciprofloxacin. Presence of beta-lactamases

### Isolate collection and susceptibility testing

The network laboratories were asked to send *N. gonorrhoeae* isolates from the samples tested for antimicrobial susceptibility between April 2014 and December 2015 to the CL for extended and comparative susceptibility testing (further labelled as isolates). There were no criteria used to preselect isolates that should be sent to CL. The sample identification number was used to link isolates to samples. For all received isolates, we confirmed *N. gonorrhoeae* by using a combination of culture on non-selective agar medium, rapid oxidase production assays and determining the presence of Gram-negative diplococci using microscopy and the Phadebact Monoclonal GC OMNI Test (Pharmacia Diagnostics, Piscataway, NJ, USA). Susceptibility testing was performed and MIC were detected by using Etest (bioMérieux SA, Marcy-l’Étoile, France) according to the manufacturer’s instructions for ceftriaxone, cefixime, azithromycin, penicillin and ciprofloxacin. To define resistance, we used the criteria of the European Committee on Antimicrobial Susceptibility Testing EUCAST 4.0 (2014) [24]. The presence of beta-lactamase enzyme production, which provides high-level resistance to penicillins, was detected by using the nitrocefin test (BBL DrySlide™, Becton, Dickinson, NJ, USA). Isolates testing positive for beta-lactamase were defined as penicillinase-producing *N. gonorrhoeae* (PPNG).

The working stock of *N. gonorrhoeae* isolates was stored at −80 °C. *N. gonorrhoeae* strains ATCC 49226 and WHO-reference strains G, K, M, O and P were used with each batch of Etest as quality controls [25]. Ceftriaxone and cefixime were tested twice when the MIC was ≥0.125 mg/L. Isolates tested in the CL were characterized by their resistance patterns.

An overview of the analysis of isolates tested in CL is given in Table 1. Note that a direct comparison of historical German data with current GORENET data was not possible because different methods for isolate collection were in place before GORENET was rolled out and the geographical coverage was different.

For categorical variables we calculated percentages and, for continuous variables, medians together with interquartile ranges (IQR) were determined. Percentages were compared by Chi-squared or Fisher’s exact tests, and medians were compared with the Wilcoxon-Mann-Whitney test, where applicable. The Kruskal-Wallis test was used to compare continuous variables between more than two categories. Significance level was set at a *p*-value < 0.05.

## Results

Of the 100 laboratories that were interested in participating in GORENET, 31 were selected for recruitment, and 23 agreed and reported data to GORENET. The reasons for declining participation in GORENET were not using the Etest for *N. gonorrhoeae* susceptibility testing (*n* = 5) and too much time and effort needed for participation (*n* = 3).

### Continuous routine data collection

Twenty-three participating laboratories reported to the RKI in total 1654 *N. gonorrhoeae* samples tested for susceptibility. Of them, 727 were collected from April to December 2014, and 927 were collected from January to December 2015. Overview of the reported samples is given in Table 1.

The number of reported samples varied between 2 and 305 per laboratory, with a median of 43 samples (IQR 24–86). In total 47.1% of the samples were reported by three laboratories, which provided information on 209 (in Hamburg), 265 (in Berlin) and 305 (in North Rhine-Westphalia) samples, respectively.

**Fig. 1:**
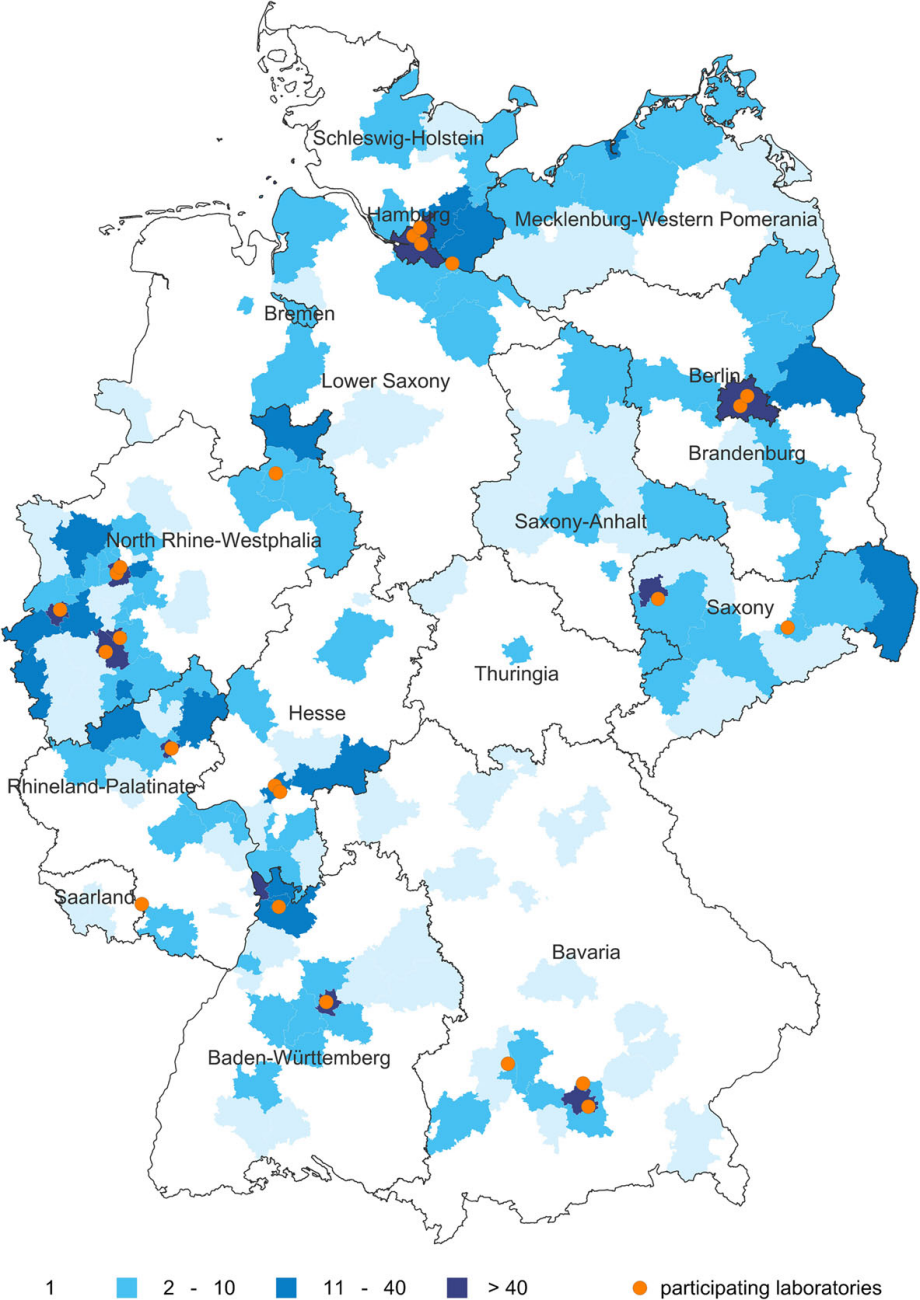
Distribution of *N. gonorrhoeae* samples by district in Germany. One thousand, six hundred and fifty-four samples from April 2014 to December 2015 (1366 defined by district code of the patient, 288 defined by district code of the laboratory). Map developed with RegioGraph Software

The number of reported *N. gonorrhoeae* samples varied by administrative district between 1 and 209 (Fig. 1). The three laboratories submitting the majority of the data were located in the areas with >50 samples.

Central and northern Germany were represented equally. Data from southern Germany mostly originated from the larger cities.

**Fig. 2:**
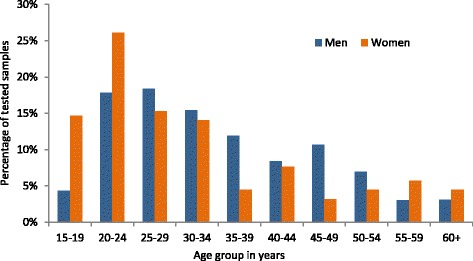
*N. gonorrhoeae* samples tested in participating laboratories, by age group and gender, *n* = 1642

Overall, 90.0% of samples tested for susceptibility in network laboratories were from men; 9.5% were from women and in nine samples, information on gender was not available. The median age of tested men and women was 33 (IQR 25–44) and 27 (IQR 22–40) years respectively (*p*-value < 0.001). The distribution of *N. gonorrhoeae* susceptibility testing by age group and gender is displayed in Fig. 2.

Most tested samples from men were from urethral (96.1%) and rectal swabs (1.7%), while among women samples came from predominately endocervical or vaginal swabs (84.3%). Most of the samples tested from men were ordered by urologists (74.4%) and from women by gynaecologists (79.7%). Distribution by gender (*p*-value = 0.25), age (*p*-value = 0.28 for men and *p*-value = 0.87 for women), tested material (*p*-value = 0.15 for men and p-value = 0.07 for women) and ordering specialist (*p*-value = 0.63 for men and *p*-value = 0.73 for women) did not differ between the years 2014 and 2015.

### Isolate collection and susceptibility testing

From the recruited 23 laboratories submitting information on samples tested for *N. gonorrhoeae* susceptibility, 16 sent isolates to the CL. We received 261 viable isolates collected between April and December 2014 and 276 viable isolates collected between January and December 2015. It was determined that 91.4% of isolates were from men, 8.4% were from women, and for one of the samples the gender was unspecified. The median age was 33 (IQR 26–43) for isolates from men and 28 (IQR 23–41) for isolates from women. These 537 isolates were tested for susceptibility in the CL. An Overview of the isolates tested in CL is given in Table 1. The results of the AMR testing of all isolates are summarized in Table 2. The percentage of resistant, intermediate and susceptible isolates did not significantly differ by age or gender.

**Table 2:** Number and percentage of *N. gonorrhoeae* isolates testing susceptible, intermediate and resistant against cefixime, ceftriaxone, azithromycin, penicillin and ciprofloxacin, *n* = 261 for year 2014 and *n* = 276 for year 2015, *p-value < 0.05

	Susceptible		Intermediate		Resistant	
	Number (percentage, %)		Number (percentage, %)		Number (percentage, %)	
	2014	2015	2014	2015	2014	2015
Cefixime	256 (98.1)	272 (98.6)	∕	∕	5 (1.9)	4 (1.4)
Ceftriaxone	261 (100)	276 (100)	∕	∕	0 (0)	0 (0)
Azithromycin	142 (54.4)	171 (62.0)	88 (33.7)	78 (28.3)	31 (11.9)	27 (9.8)
Ciprofloxacin*	73 (28.0)	114 (41.3)	0 (0)	1 (0.4)	188 (72.0)	161 (58.3)
Penicillin*	27 (10.3)	40 (14.5)	158 (60.5)	184 (66.7)	76 (29.1)	52 (18.8)

**Fig. 3:**
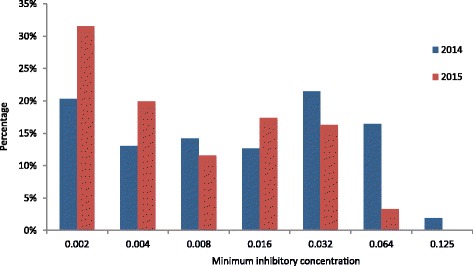
Distribution of minimum inhibitory concentrations for ceftriaxone, *n* = 261 for year 2014 and *n* = 276 for year 2015

No resistance to ceftriaxone (MIC >0.125 mg/L) was detected in 2014 or 2015 (Fig. 3). In 2014, two isolates showed MICs at the estimated breakpoint of 0.125 mg/L. One of these isolates displayed further resistance to cefixime, azithromycin and ciprofloxacin and showed an intermediate test result to penicillin (isolate 1, Table 3). Another isolate displayed resistance to ciprofloxacin and penicillin, while it was intermediate to azithromycin and had a MIC value at the breakpoint for cefixime (isolate 10, Table 3).

**Table 3:** Minimum inhibitory concentrations (MIC) of cefixime, ceftriaxone, azithromycin, ciprofloxacin and penicillin for isolates with resistance to cefixime or with MICs at the breakpoint for resistance to cefixime (*n* = 24)

	Minimum inhibitory concentration (MIC)				
Isolate	Cefixime	Ceftriaxone	Azithromycin	Ciprofloxacin	Penicillin
1	0.25	0.125	0.75	32	1
2	0.19	0.047	1	0.25	32
3	0.19	0.094	0.75	32	3
4	0.19	0.023	0.19	32	0.75
5	0.19	0.047	0.125	32	0.75
6	0.19	0.032	0.19	32	1.5
7	0.19	0.032	0.094	12	0.75
8	0.19	0.032	0.19	16	1
9	0.19	0.023	0.125	16	0.75
10	0.125	0.125	0.5	32	2
11	0.125	0.064	1	32	1
12	0.125	0.064	1	32	2
13	0.125	0.032	0.19	32	0.75
14	0.125	0.032	0.19	32	0.75
15	0.125	0.032	0.19	32	0.5
16	0.125	0.032	0.125	32	1.5
17	0.125	0.023	0.125	32	0.38
18	0.125	0.023	0.19	32	0.75
19	0.125	0.023	0.19	32	0.5
20	0.125	0.023	0.125	16	0.75
21	0.125	0.023	0.094	12	0.75
22	0.125	0.016	0.25	32	0.75
23	0.125	0.012	0.125	12	0.38
24	0.016	0.006	256	12	32

**Fig. 4:**
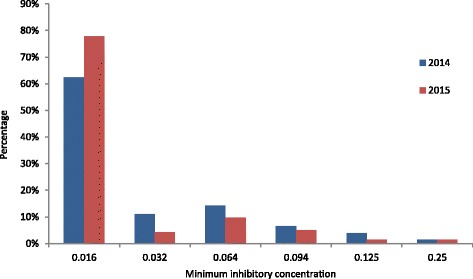
Distribution of minimum inhibitory concentration for cefixime, *n* = 261 for year 2014 and *n* = 276 for year 2015

Altogether, 1.9% (*n* = 5) in 2014 and 1.4% (*n* = 4) in 2015 of the isolates displayed resistance (MIC >0.125 mg/L) to cefixime. The majority of isolates (62.5% in 2014 and 77.9% in 2015) showed low MICs of ≤0.016 mg/L to cefixime (Fig. 4). In 2014, 3.8% (*n* = 10) and in 2015, 1.4% (*n* = 4) of the isolates had a MIC of 0.125 mg/L at the estimated breakpoint.

All isolates with resistance to cefixime displayed resistance to ciprofloxacin. Three of the nine cefixime-resistant strains also showed resistance to azithromycin. No cefixime-resistant strain was susceptible to penicillin (3 resistant, 6 intermediate).

One cefixime-resistant isolate (MIC 0.25 mg/L) displayed additional resistance against azithromycin and ciprofloxacin, intermediate susceptibility against penicillin and showed reduced susceptibility to ceftriaxone at the breakpoint of MIC 0.125 mg/L (isolate 1, Table 3).

All isolates displaying resistance to cefixime were from men.

**Fig. 5:**
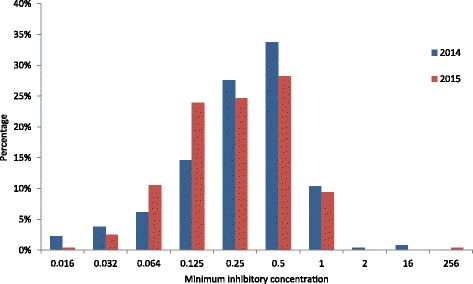
Distribution of minimum inhibitory concentration for azithromycin, *n* = 261 for year 2014 and *n* = 276 for year 2015

A total of 11.9% (2014) and 9.8% (2015) of the isolates showed resistance against azithromycin (MIC >0.5 mg/L). In addition, there was a high percentage of *N. gonorrhoeae* strains with intermediate susceptibility (33.7% in 2014 and 28.3% in 2015) and the MIC distribution of the susceptible strains appeared closer to intermediate breakpoint (MIC >0.38 mg/L) (Fig. 5). The MICs of resistant strains were mostly low and showed a distribution concentrating around the breakpoint (MIC >0.5 mg/L).

In 2015, one isolate displayed high-level resistance to azithromycin (MIC >256 mg/L). This isolate also showed high-level resistance to penicillin and resistance to ciprofloxacin, but was susceptible to ceftriaxone and cefixime.

The percentage of strains with resistance to ciprofloxacin (MIC >0.06 mg/L) was 72.0% in 2014 and 58.3% in 2015 (Table 2)**.**

Overall, 29.1% of the isolates in 2014 and 18.8% in 2015 displayed resistance to penicillin. In addition, there was a very high rate of intermediate *N. gonorrhoeae* strains (Table 2).

Nitrocefin testing for the detection of beta-lactamase activity in *N. gonorrhoeae* was performed in 83.5% (*n* = 218) of isolates in 2014. All 276 isolates (100%) were tested for beta-lactamase activity in 2015. High-level plasmid-mediated resistance against penicillin (penicillinase producing *N. gonorrhoeae*, PPNG) was found in 25% of all tested strains in 2014 and in 14% of the strains in 2015.

## Discussion

Using GORENET we aimed at two targets concerning gonococcal infections: data collection on disease epidemiology and monitoring of resistance patterns with unified methodology.

We were able to implement a nationwide data collection of all performed *N. gonorrhoeae* susceptibility testing in the participating laboratories. Routine data collection on all performed *N. gonorrhoeae* susceptibility tests together with epidemiological information, like age and gender, was not in place until GORENET surveillance.

Before starting GORENET, the CL collected isolates from a range of laboratories. This pre-existing network was expanded within GORENET, and the number of collected isolates increased substantially.

We were able to set up electronic data collection for all samples tested for *N. gonorrhoeae* susceptibility in the participating laboratories. Timely transmitted data are a good tool for monitoring *N. gonorrhoeae* susceptibility dynamics. Due to data protection issues, the collected epidemiological information was limited and important information, such as data regarding the transmission route, therapeutic regimen and therapeutic success, could not be gathered.

We reached a relatively even geographical representation of all regions, but the coverage in central and southern Germany should be increased further.

In routine data collection from the network laboratories, we found that over 90% of all samples tested for *N. gonorrhoeae* susceptibility were from men, similar to several other European countries [22]. This percentage was 84% in previous studies in Germany [15], and we can assume that at least half of all isolates from men are attributable to men having sex with men (MSM), comparable to other European countries [22]. Nevertheless, women might still be underrepresented in our sample. This seems possible because men are more often symptomatic and might therefore be tested more often than women [14]. We aim to collect data on transmission routes from physicians to better interpret the collected data.

We also found that the median age of tested men was slightly higher than that of tested women. Again, Euro-GASP data and other previous data from Germany have reported findings [18, 21, 22] similar to our results. In countries that reported the risk of transmission, the proportion of men aged >25 years was higher among MSM than among heterosexual men. This might explain the higher median age of the tested men in our sample. As *N. gonorrhoeae* is not reportable in Germany, we were not able to compare whether the *N. gonorrhoeae* AMR test distribution by age corresponded to the age groups most affected by gonorrhoea.

We exclusively analysed susceptibility data from the CL testing because presently Germany lacks a standard operation protocol and regular quality assurance for *N. gonorrhoeae* testing for all laboratories. From a cross-sectional survey among laboratories performing *N. gonorrhoeae* diagnostics we know that 55% of all laboratories (76% of private laboratories) are accredited and 19% are certified [23]. However, due to the use of different methods, standards and test panels it is unknown if there are substantial differences in quality of testing, and GORENET will be a useful tool for quality assurance in the future.

Susceptibility testing in the CL enabled the monitoring of *N. gonorrhoeae* antimicrobial resistance detected by a unified methodology. Age and gender distribution of the tested isolates did not differ from all samples tested for susceptibility in the participating laboratories.

No resistance to ceftriaxone was detected in 2014 and 2015 in isolates collected with GORENET, and only two isolates showed MICs at the estimated breakpoint of 0.125 mg/L.

Previous German publications also did not show notable levels of ceftriaxone resistance or ESC resistance [18–21] in Germany. However, within the Euro-GASP collection, there was one exception in 2011, when 6.5% of German strains were resistant against ceftriaxone. In the following years 2012–2014, there were no notable levels of ceftriaxone resistance observed [18, 21, 22] in Germany by the Euro-GASP surveillance.

Currently, parenterally administered ceftriaxone remains an effective treatment option for gonorrhoea in Germany. Similar data have been published from surveillance systems in other countries [13, 26].

The percentage of strains with resistance to cefixime remained moderate within the GORENET (<2%), and low MIC values were predominant. German Euro-GASP data from 2014 correspond to this GORENET observation. In 2014 no cefixime-resistant isolate from Germany was detected within the Euro-GASP surveillance [22]. Compared with previous German data from Euro-GASP, this is a decrease from the years 2009–2013, when resistance rates from 5.7% to 12.9% were detected [22]. The decreasing number of strains resistant to cefixime could be observed not only in GORENET: European Euro-GASP data from 2014 and surveillance reports from the United Kingdom, the United States and Canada showed the same trend to less ESC-resistant isolates [26–29]. The decrease in gonococcal resistance to cefixime in 2014/15 suggests that clinicians in Germany may have avoided prescribing this antibiotic as a first-line treatment after the new therapy guidelines were published. However, changes in resistance patterns develop incrementally and are usually not detected so fast.

A presumptive explanation for this observation is the eradication of previously undetected reservoirs [27, 30]. Especially extragenital infections, which are oftentimes asymptomatic, difficult to culture and difficult to treat, are a constant reservoir for the spread of gonorrhoea [9, 30, 31]. Due to regular use of highly sensitive molecular diagnostic tests, like nucleic acid amplification tests (NAAT), in the routine diagnostic, detection of gonococcal infections was improved. Extragenital infections and subclinical urogenital infections are therefore more often confirmed and can be successfully treated [31]. The adjustment of treatment guidelines for ceftriaxone as first-line therapy seems to provide here an additional benefit. Sufficient treatment of these infections prevents a selection of resistant clones and might be a reason for the decrease of cefixime-resistant gonococci [27, 28, 30].

Molecular typing studies, performed with *N. gonorrhoeae* multiantigen sequence typing (NG-MAST, http://www.ng-mast.net) [32], showed one sequence type (ST1407), which was the most frequently observed sequence type associated with ESC- and multi-resistance [33–35]. Therefore, a further reason for the decrease of cefixime resistance could be a replacement of this multidrug-resistant *N. gonorrhoeae* clone ST1407 by clones with different resistance patterns within the infected population [27]. This could be an effect of the sufficient treatment or, as the Euro-GASP authors pointed out, be caused by impaired reinfection with the same clone due to a partial immunity and needs to be evaluated by future typing studies [27]. Additional monitoring and a molecular typing study within GORENET in the next years are therefore intended.

Multidrug resistance was not detected regularly in GORENET. Only one cefixime-resistant isolate displayed further resistance to azithromycin and ciprofloxacin, intermediate susceptibility to penicillin and a reduced susceptibility to ceftriaxone at the breakpoint MIC of 0.125 mg/L. Nevertheless, the combination of resistances is particularly alarming and should be monitored further.

A high prevalence of resistance was detected for azithromycin. Although we observed mostly resistance near the breakpoint (MIC >0.5 mg/L), this trend is concerning. In addition, there was a high rate of intermediate *N. gonorrhoeae* strains: 40–45% of the strains were not fully susceptible to azithromycin. Germany’s data from previous years [22] shows that the level of azithromycin resistance was mostly under 5%. The first-line use of azithromycin is very common in STI treatment regimes, especially as a syndromic treatment before or without confirmation of the pathogenic agent. This might explain the increase in azithromycin resistance in the last several years, but data on prescriptions in Germany are not published.

In 2015, we detected the first case of a high-level azithromycin resistant *N. gonorrhoeae* strain in Germany, with a MIC >256 mg/L. This isolate was susceptible to ceftriaxone and cefixime but showed high-level resistance to penicillin and resistance to ciprofloxacin. As rising resistance rates to azithromycin are increasingly observed globally [22, 26] and high-level azithromycin resistance is reported worldwide [35–43], azithromycin is not suitable for first-line treatment. If azithromycin is used as a single-drug treatment in cases of severe penicillin/cephalosporin anaphylaxis, susceptibility testing prior to treatment should be performed.

According to current treatment guidelines, dual therapy with ceftriaxone and azithromycin is recommended, using two antimicrobial agents with different mechanisms of action [5, 44]. Currently, there is no alternative to this dual treatment regime. Ultimately, we will need to discuss whether we have to abandon dual therapy with azithromycin if the trend of increasing azithromycin resistance remains in the years to come [9, 45].

The resistance rates to ciprofloxacin were constantly high in Germany, although a drop was detectable in the surveillance period. A high prevalence of resistance to ciprofloxacin has also been found in Europe and worldwide since the late 1990s [22]. The drug is therefore not recommended for therapeutic use in Germany.

Resistance to penicillin has been prevalent for many decades worldwide. Nearly 25% of all isolates displayed resistance to penicillin and an additional 64% showed intermediate susceptibility in the GORENET surveillance data. Accordingly, approximately 90% of the strains were not fully susceptible to penicillin in Germany. High-level plasmid-mediated resistance to penicillin was also regularly observed but decreased within the surveillance period from 25.9% to 14%, an observation that requires further monitoring.

We have several limitations in our data. First, within the GORENET data collection scheme, we were unable to collect more detailed epidemiological information, such as the risk of transmission, special symptoms, therapy strategies and treatment success rates. Usually, laboratories in Germany have very limited epidemiological information available, provided by the physicians in charge, and need to treat these data confidentially due to strict data-protection regulations.

Second, our data analysis was limited by the small number of isolates collected compared to the overall population of Germany.

Third, we cannot exclude selection bias in our data, because we recruited laboratories based on their catchment area, number of tested *N. gonorrhoeae* samples and use of the Etest from a pool of laboratories that expressed an interest to participate. However, with selected and established network laboratories, which forwarded all of their received isolates, we diminished a collection bias from laboratories being more prone to submit isolates with interesting resistance patterns.

## Conclusions

Using GORENET, we were able to implement a nationwide collection of all performed *N. gonorrhoeae* susceptibility data in the participating laboratories and increase the number of collected isolates retested at the CL for confirmation and quality assurance. The majority of susceptibility tests are performed among young men. More detailed epidemiological information would be beneficial.

The resistance rate to ceftriaxone remains low in Germany. Therefore, ceftriaxone is still an appropriate treatment for gonorrhoea at present. In 2014 and 2015, we found low resistance rates for cefixime in Germany. However, this needs further monitoring. Resistance to azithromycin is common and should continue to be monitored in the future. Except for a small decrease in AMR towards ciprofloxacin and penicillin, no substantial changes in the susceptibility patterns between 2014 and 2015 could be detected.

In conclusion, GORENET as a gonococcal antimicrobial surveillance in Germany is highly needed. Current data and ongoing collection of data will be used to update national treatment guidelines and, if necessary, implementation of future prevention measures.

To continue to monitor *N. gonorrhoeae* susceptibility, particularly against ESC and azithromycin, the yearly number of isolates tested in the CL should be substantially increased. Molecular surveillance of the circulating strains is important for monitoring the current situation, the evolving resistances and the transmission networks.

Surveillance of susceptibility is essential to ensure efficient patient management and keep gonorrhoea a treatable disease.

## Abbreviations

AMR: Antimicrobial resistance
ARS: Antibiotic resistance surveillance programme
ATCC: American Type Culture Collection
CDC: Centers for Disease Control and Prevention
CL: Consiliary Laboratory for gonococci
ECDC: European Centre for Disease Prevention and Control
ESC: Extended spectrum cephalosporin
EUCAST: European Committee on Antimicrobial Susceptibility Testing
Euro-GASP: European Gonococcal Antimicrobial Surveillance Programme
GORENET: Gonococcal Resistance Network
i.m.: Intramuscular
i.v.: Intravenous
IQR: Interquartile ranges
MSM: Men having sex with men
*N. gonorrhoeae*: *Neisseria gonorrhoeae*
NAAT: nucleic acid amplification test
NG MAST: *Neisseria gonorrhoeae* multiantigen sequence typing
PPNG: Penicillinase producing *N. gonorrhoeae*
RKI: Robert Koch Institute
ST: Sequence type
STI: Sexually transmitted infection
WHO: World Health Organization
